# Supplementary material for: Complexes of Sodium Pectate with Nickel for Hydrogen Oxidation and Oxygen Reduction in Proton-Exchange Membrane Fuel Cells
Source: Int J Mol Sci. 2022 Nov 17;23(22):14247. doi: 10.3390/ijms232214247 (PMC9695899; doi:10.3390/ijms232214247)
Supplement: Supplementary file 1 [file ijms-23-14247-s001.zip › ijms-1980851-supplementary.pdf]

International Journal of *Molecular Sciences*

MDPI

Supplementary Material

Complexes of Sodium Pectate with Nickel for Hydrogen Oxidation and Oxygen Reduction in Proton-Exchange Membrane Fuel Cells

Irek R. Nizameev[1,2], Danis M. Kadirov[1,3], Guliya R. Nizameeva[1,3], Aigul' F. Sabirova[1], Kirill V. Kholin[1,3], Mikhail V. Morozov[2], Lyubov' G. Mironova[1], Salima T. Minzanova[1], Oleg G. Sinyashin[1], and Marsil K. Kadirov[1,3,*]

[1] Arbuzov Institute of Organic and Physical Chemistry, FRC Kazan Scientific Center, Russian Academy of Sciences, Kazan 420088, Russia
[2] Kazan National Research Technical University named after A.N. Tupolev - KAI, Kazan 420111, Russia
[3] Kazan National Research Technological University, Kazan 420015, Russia
[*] Correspondence: kamaka59@gmail.com; Tel.: +7(927) 417-45-59

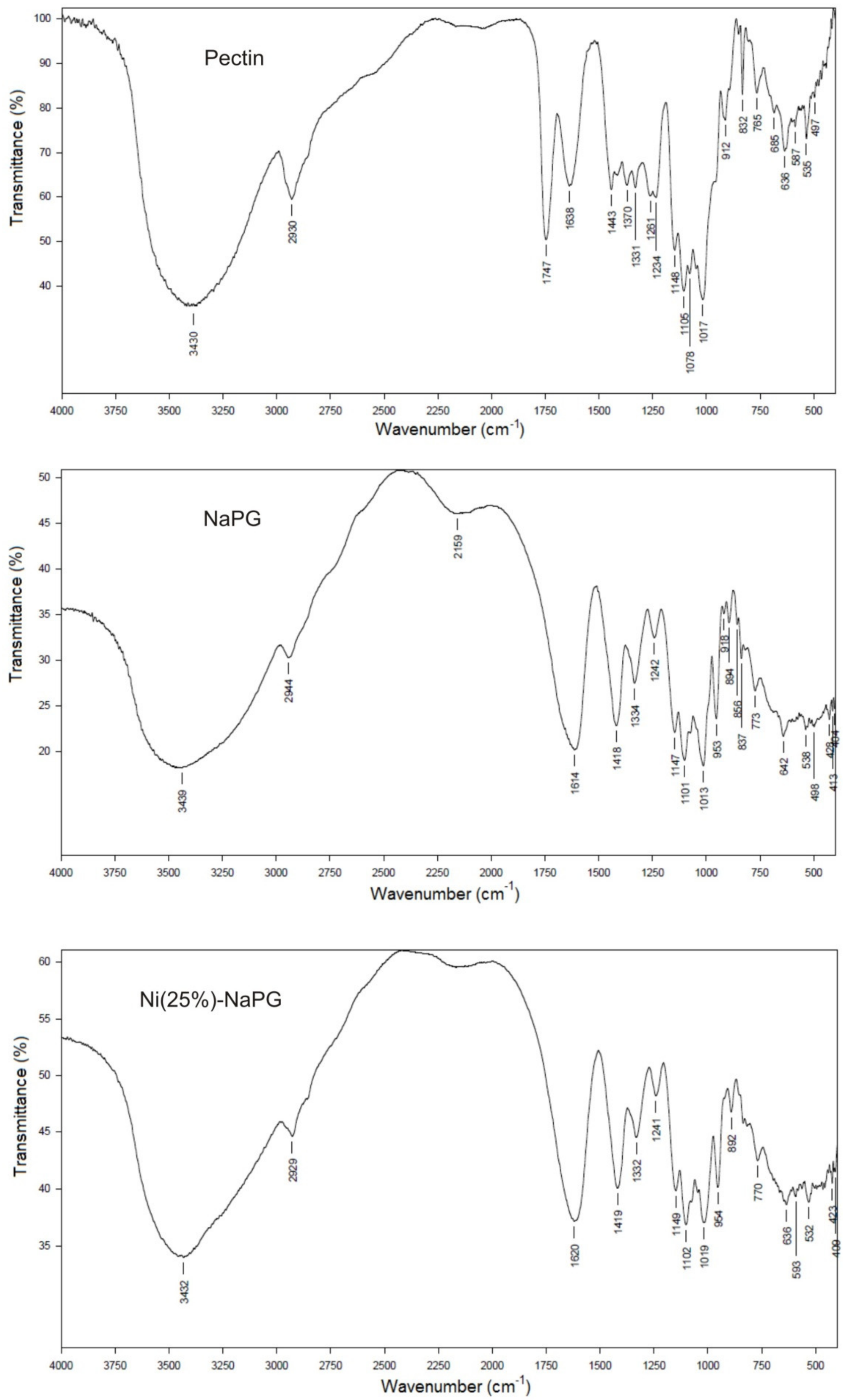

Figure S1. IR spectra of the pectin, sodium polygalacturonate (NaPG) and sodium polygalacturonate complex [Ni(25%)-NaPG] with 25% substitution of sodium with nickel.

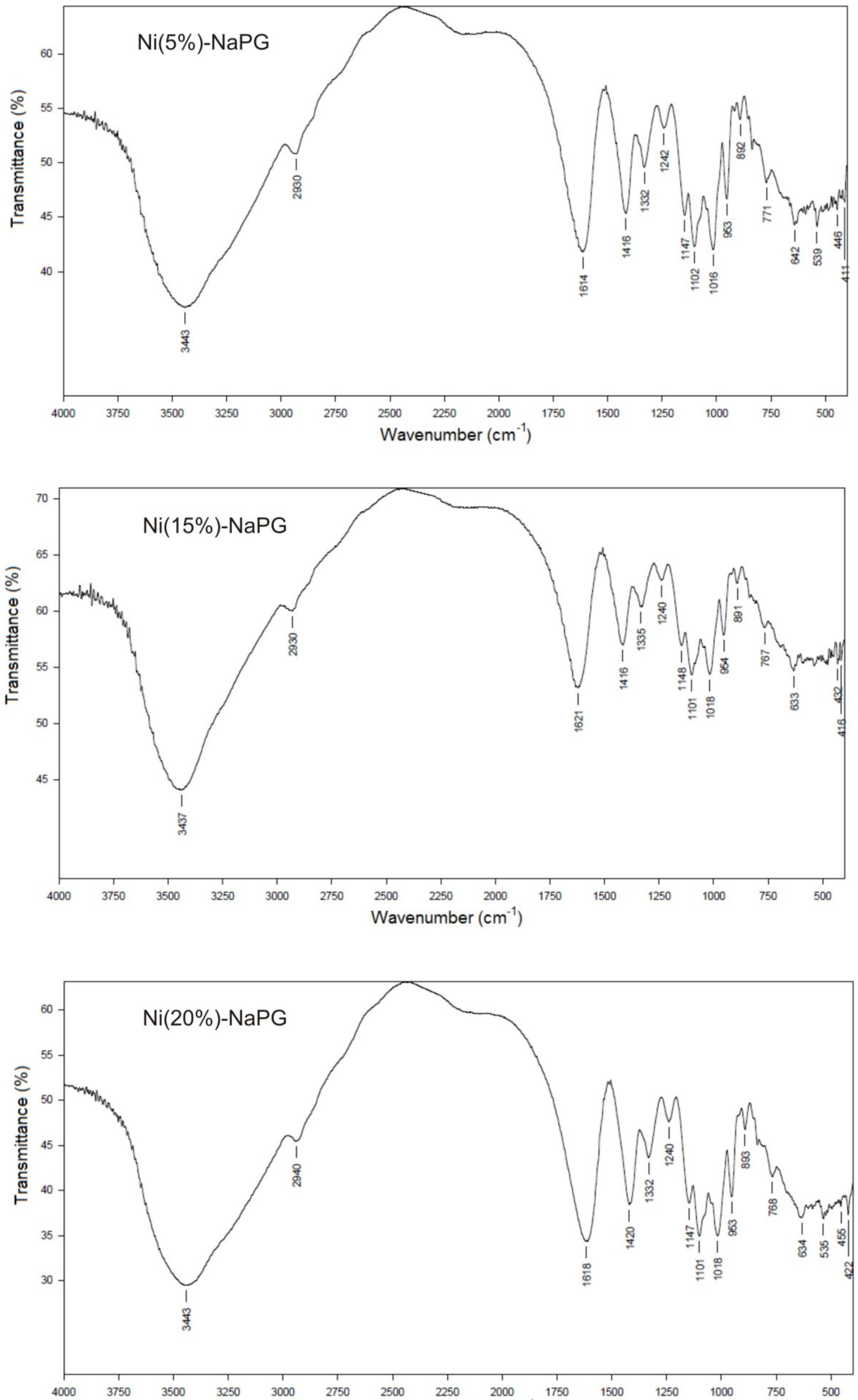

Figure S2. IR spectra of the sodium polygalacturonate complexes Ni(5%)-NaPG, Ni(15%)-NaPG, Ni(20%)-NaPG.

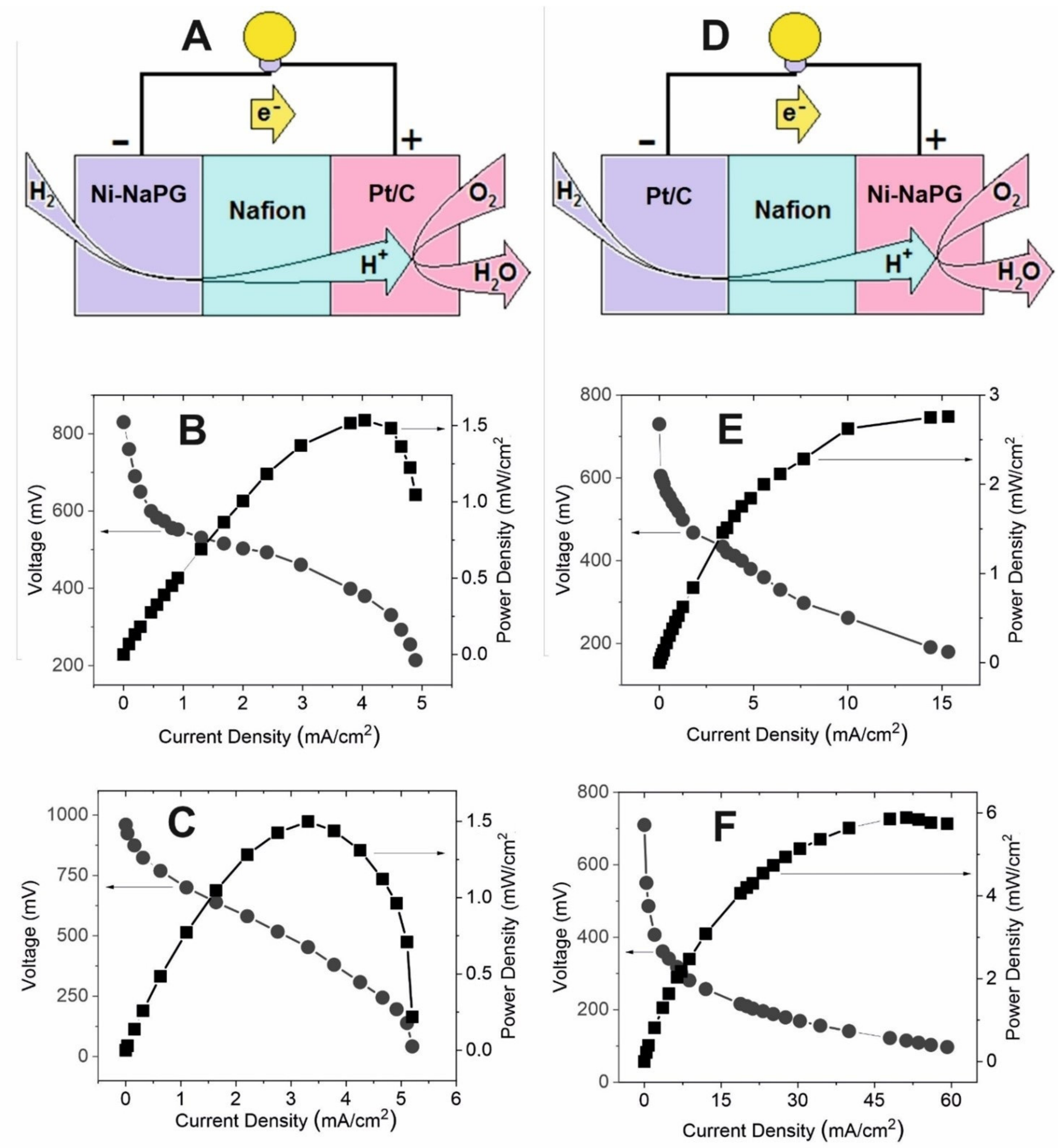

Figure S3. Representations (A, D) of the H_2/O_2 PEMFC and polarization and power curves at 80ºC for MEAs Ni(15%)-NaPG/Nf/Pt (B),Ni(25%)-NaPG/Nf/Pt[a] (C),Pt/Nf/Ni(15%)-NaPG (E), Pt/Nf/Ni(25%)-NaPG[b] (F).

[a] FromM.K. Kadirov, S.T. Minzanova, I.R. Nizameev, M.N. Khrizanforov, L.G. Mironova, K.V. Kholin, D.M. Kadirov, E.S. Nefed‘ev, M.V. Morozov, A.T. Gubaidullin, Yu.H. Budnikova, O.G. Sinyashin, *ChemistrySelect,* 2019, *4*, 4731-4734.

[b]From Kadirov, M. K., Karasik, A. A., Nizameev, I. R., Strelnik, I. D., Kholin, K. V., Kadirov, D. M.,Ismaev, T.I., Budnikova, Y.H.,Sinyashin, *Energy Technology*, 2018. *6*(6), 1088-1095.

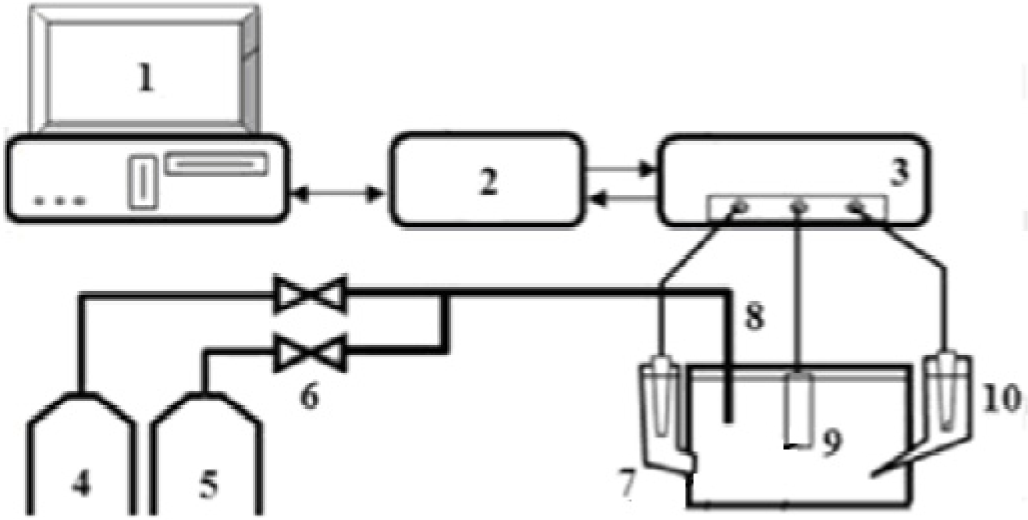

Figure S4. The scheme of electrochemical set-up. 1- computer, 2 – potentiostat Elins P-20x, 3 – rotating disc electrode – Basi RDE-2, 4 – vessel with inert gas (Ar), 5 – vessel with oxygen, 6 – valve of microadjusing, 7 – counter electrode, 8 – capillary for gas introducing, 9 – working electrode, 10 – reference electrode.

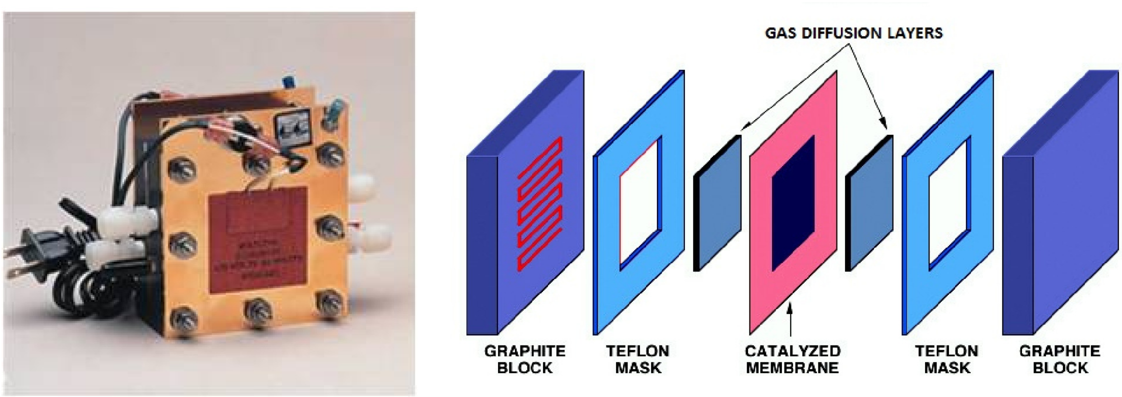

Figure S5. Scheme and appearance of the standard PEMFC used in the tests.
